# Editorial: Improving Extracorporeal Life Support Outcomes in Children

**DOI:** 10.3389/fped.2019.00140

**Published:** 2019-04-17

**Authors:** Hitesh S. Sandhu, James D. Fortenberry, Graeme MacLaren

**Affiliations:** ^1^Division of Pediatric Critical Care, Department of Pediatrics, University of Tennessee Health Science Center, Memphis, TN, United States; ^2^Division of Critical Care, Department of Pediatrics, Emory University School of Medicine, Children's Healthcare of Atlanta, Emory University, Atlanta, GA, United States; ^3^Cardiothoracic ICU, National University Hospital, Singapore, Singapore; ^4^Paediatric ICU, Department of Paediatrics, University of Melbourne, Melbourne, VIC, Australia

**Keywords:** extracorporeal life support, long term outcomes, collaborative research, ECPR = extracorporeal CPR, multi-organ failure, hybrid ECLS, ECLS for Glenn and Fontan circulation

Extracorporeal life support (ECLS, also known as extracorporeal membrane oxygenation or ECMO) emerged in the 1970s as a potentially useful life-support therapy for refractory cardiac or respiratory failure in neonatal and pediatric patients (1–3). Following a successful randomized controlled trial of ECLS in neonatal respiratory failure in the mid-1990s (4, 5), ECLS became a standard of care in neonatal and pediatric intensive care medicine.

In order to continue refining the use of ECLS in pediatrics, there have to be sustained efforts on improving long-term outcomes after ECLS and reducing complications. This may be best achieved by a multipronged strategy at the institutional level coupled to collaborative multicenter research, use of innovative clinical strategies, and use of the Extracorporeal Life Support Organization (ELSO) Registry and other databases to guide decision making.

In this collection of articles commissioned by the Journal, we collectively explore strategies to improve long term outcomes after ECLS and outline methods for future research and innovation in the field of pediatric and neonatal ECLS.

To ensure a high level of competency in ECLS use at an institutional level, there has to be commitment to improving the dissemination of information, standardization of training, practice, and education of ECLS physicians and specialists. Raffaeli et al. describe their experience in initiating a new ECLS program. They use the experience of established centers and the guidelines supplied by ELSO to establish a comprehensive training program over a 4 year period to increase the knowledge and comfort level of ECLS providers. They use traditional didactic and water drill sessions, followed by high definition simulation training to establish a successful ECLS program.

While the use of pediatric ECLS has been stable over time, neonatal ECLS volumes have declined over the last decade. The Adult ECMO collaborative, International ECMO network (ECMOnet) (6, 7) has demonstrated the value of pooling resources between centers to achieve meaningful research. Similarly, pediatric and neonatal centers may benefit from coming together to pool patients and resources to answer the most important questions regarding this population. Bembea et al. present the history of pediatric ECMO research and the barriers to clinical research in pediatric ECMO. They discuss the opportunities for multicenter, multi-network research collaboration for future pediatric ECMO studies.

The use of ECLS in patients with multi-organ failure facilitates the presence of the extracorporeal circuit as a stable platform for providing multi-organ support. Canter et al. describe supporting critically ill patients with multi-organ failure while waiting for organ recovery or as a bridge to transplant. They explore the indications, different techniques and results in the use of CRRT, therapeutic plasma exchange, leukopheresis, adsorption therapy, and extracorporeal liver support.

While more complex patients are being successfully supported with ECLS, a significant number of complications are associated with its use. This has led to increased interest in the evaluation of long-term outcomes and also measures to prevent complications. IJsselstijin et al. propose that follow up programs after ECLS should be the standard of care for all patients. They successfully realized this in their own country. ECLS follow-up should be structured, multidisciplinary and standardized to identify and treat any long-term sequelae identified.

There is increasing use of ECLS during cardiac arrest, also known as extracorporeal cardiopulmonary resuscitation (ECPR). Laussen and Guerguerian describe the phases of ECPR and the resources, personnel, and protocols needed to start and sustain a successful ECPR program. They discuss the reasons for the better outcomes with ECPR in pediatric cardiac patients compared to conventional CPR. They stress the need for regular simulation and debriefing sessions to maintain quality and outcomes.

Historically, the use of ECLS in children with Glenn and Fontan physiology has been challenging due to their unique physiology. Bacon et al. analyze recent studies of ECLS in these patients. They outline strategies to ensure adequate venous drainage and avoidance of cerebral injury in Glenn physiology. In patients with Fontan physiology, they recommend higher ECLS flow rates to decompress the heart and provide adequate circulatory support. They define the three stages of Fontan failure and specific treatment strategies for each phase.

Lastly, Maul et al. explore adapting ECLS technology in novel ways to support patients in specific clinical circumstances, including the use of hybrid ECLS systems, alternative cannulation strategies, paracorporeal lung devices, and extracorporeal CO_2_ removal devices. Given the shortage of organ donors worldwide and the emerging need to provide prolonged organ support to children awaiting transplantation, it is crucial to adapt existing technology in this manner.

These excellent contributions summarize current state-of-the-art ECLS use in children, from instituting a neonatal ECLS program to developing a resource intensive ECPR program. The collected work explores the cutting edge of existing technology and adaptive use of ECLS. The articles include a suggested framework for a research collaborative and long term follow-up to achieve the goal of reducing complications and improving the long term outcomes. We hope that readers of the Journal find this series a valuable spur to new research and innovation in extracorporeal support.

## Author Contributions

HS, GM, and JF contributed conception and design of the editorial. HS wrote the first draft of the manuscript. GM and JF wrote sections of the manuscript. All authors contributed to manuscript revision, read, and approved the submitted version.

### Conflict of Interest Statement

The authors declare that the research was conducted in the absence of any commercial or financial relationships that could be construed as a potential conflict of interest.
